# Early Introduction and Community Transmission of SARS-CoV-2 Omicron Variant, New York, New York, USA

**DOI:** 10.3201/eid2902.220817

**Published:** 2023-02

**Authors:** Dakai Liu, Yexiao Cheng, Hangyu Zhou, Lulan Wang, Roberto Hurtado Fiel, Yehudah Gruenstein, Jean Jingzi Luo, Vishnu Singh, Eric Konadu, Keither James, Calvin Lui, Pengcheng Gao, Carl Urban, Nishant Prasad, Sorana Segal-Maurer, Esther Wurzberger, Genhong Cheng, Aiping Wu, William Harry Rodgers

**Affiliations:** NewYork-Presbyterian Queens Hospital, Flushing, New York, USA (D. Liu, J.J. Luo, V. Singh, E. Konadu, K. James, C. Lui, P. Gao, C. Urban, N. Prasad, S. Segal-Maurer, W.H. Rodgers)**;**; Chinese Academy of Medical Sciences & Peking Union Medical College, Beijing, China (Y. Cheng, H. Zhou, A. Wu);; Suzhou Institute of Systems Medicine, Suzhou, China (Y. Cheng, H. Zhou, A. Wu)**;**; University of California, Los Angeles, California, USA (L. Wang, G. Cheng);; Kaiser Permanente Health, North Valley, California, USA (R.H. Fiel)**;**; LabQ Diagnostics, Brooklyn, New York, USA (Y. Gruenstein, E. Wurzberger);; Weill Cornell Medical College, New York (J.J. Luo, C. Urban, N. Prasad, S. Segal-Maurer, W.H. Rodgers)

**Keywords:** COVID-19, SARS-CoV-2, Omicron, subclade multiple introductions, co-circulation, community transmission, household transmission, workplace transmission, New York, United States, viruses, respiratory infections, zoonoses, *Suggested citation for this article*: Liu D, Cheng Y, Zhou H, Wang L, Fiel RH, Gruenstein Y, et al. Early introduction and community transmission of SARS-CoV-2 Omicron variant, New York, New York, USA. Emerg Infect Dis. 2023 Feb [*date cited*]. https://doi.org/10.3201/eid2902.220817

## Abstract

The Omicron variant of SARS-CoV-2 has become dominant in most countries and has raised significant global health concerns. As a global commerce center, New York, New York, USA, constantly faces the risk for multiple variant introductions of SARS-CoV-2. To elucidate the introduction and transmission of the Omicron variant in the city of New York, we created a comprehensive genomic and epidemiologic analysis of 392 Omicron virus specimens collected during November 25–December 11, 2021. We found evidence of 4 independent introductions of Omicron subclades, including the Omicron subclade BA.1.1 with defining substitution of R346K in the spike protein. The continuous genetic divergence within each Omicron subclade revealed their local community transmission and co-circulation in New York, including both household and workplace transmissions supported by epidemiologic evidence. Our study highlights the urgent need for enhanced genomic surveillance and effective response planning for better prevention and management of emerging SARS-CoV-2 variants.

During the global pandemic of SARS-CoV-2, novel variants have continuously emerged (*1*). Some variants constitute an increased risk to global public health and are being monitored as variants of concern by the World Health Organization (WHO). The Omicron variant was detected from patients in Botswana and South Africa in November 2021 (*2*); it was designated as the fifth variant of concern. Since its detection, it rapidly spread across the world and became the predominant variant in several countries (*3*). Omicron has a higher number of mutations than previously detected variants. Of note, some mutations located in the receptor-binding domain (RBD) of the spike (S) protein altered the immune escape ability of Omicron (*4*–*6*). The 69–70 deletion in the S gene of Omicron can be characterized by the failure to detect the S gene using certain diagnostic tests (*7*), known as the S gene target failure (SGTF). In the context of the global dominance of Delta and Omicron, some studies defined the Omicron case as the presence of SGTF and the Delta case as the absence of SGTF in the test samples (*8*–*10*). Three studies reported the genomic, epidemiologic, and clinical analysis of early Omicron introductions (*10*–*12*); combining viral genome analysis with epidemiologic evidence supports the study of introduction and community transmission patterns of emerging viruses.

As a major cosmopolitan city, New York, New York, USA (NYC), has been at risk for multiple variant introductions of SARS-CoV-2 during the COVID-19 pandemic (*13*–*15*). Studies have shown that a patient infected with the Omicron variant attended a large indoor convention with attendees from 52 US jurisdictions and 30 foreign countries during November 19–November 21, 2021, in NYC; a total of 119 event-associated cases were identified after the investigation (*16*,*17*). In addition, some Omicron-associated mutations were detected in wastewater in NYC on November 21, 2021 (*18*). This evidence indicates that the Omicron variant was introduced to NYC early in its outbreak. Shortly after its introduction, Omicron replaced Delta as the dominant variant in NYC (https://www1.nyc.gov/site/doh/covid/covid-19-data-variants.page), suggesting potential community transmission.

In this study, we performed whole-genome sequencing for 392 Omicron viruses obtained from persons in NYC during November 25–December 11, 2021. These dates encompass the expected early Omicron introduction into the city. Using the epidemiologic and genetic data of these sequencing samples, we determined the introduction and community transmission pattern of early Omicron in NYC. This study was reviewed and approved by the NewYork-Presbyterian Queens Hospital Institutional Review Board (IRB no. 13740321).

## Methods

### Sample Collection

Being in the epicenter of the COVID-19 pandemic, NewYork-Presbyterian Queens Hospital has received 185,870 specimens for SARS-CoV-2 RNA testing by diagnostic multiplex real-time PCR since the COVID-19 pandemic started in March 2020. In addition to those specimens, we analyzed specimens collected by mobile service vans from residential communities and workplaces. We used demographics, including residential and business addresses associated with collection sites, for the epidemiology analysis. We considered households as persons at the same residential address, identical business addresses as workplace, and family members as family. We determined traveler status by the home address and traveling inquiry performed during sampling. We advised all patients testing positive or exposed patients to follow the Centers for Disease Control and Prevention (CDC) quarantine guidelines.

### Viral Genomic Next-Generation Sequencing and Bioinformatics Processing

To investigate SARS-CoV-2 mutations and variant epidemiology, we performed next-generation sequencing (NGS) on the positive specimens with real-time PCR cycle threshold (Ct) value <33 cycles and analyzed virus mutations among the specimens from our laboratory and LabQ Diagnostics (New York, New York, USA). We performed NGS by using the Illumina COVID-Seq test kit (https://www.illumina.com). We extracted viral RNA from a viral transport medium containing a nasopharyngeal swab specimen, then performed cDNA synthesis through reverse transcription using random hexamer primers. We amplified the cDNA of the viral genome by 2 separate PCR reactions and pooled the products together. The fragments underwent bead-based tagmentation to the adaptor sequences. Subsequently, the adaptor-tagged fragments underwent another round of PCR amplification. Using the purification beads, we pooled and cleaned the indexed tagged libraries. We clustered pooled libraries onto a flow cell and then sequenced on the NovaSeq 6000 sequencing system (Illumina). We used VarSeq version 2.2.2 (Golden Helix, https://www.goldenhelix.com) for sequence analysis; we used consensus sequences of these viruses as input to Nextclade version 1.10.1 (*19*) for quality control, mutation calling, and Nextstrain clade assignment. Viruses <29,000 nt in length or with Nextclade-assessed QC.overallStatus below good were considered low quality and removed.

### Phylogenetic Analysis

To investigate the genetic relationship between Omicron viruses in NYC, we constructed a genotype network of all sequenced Omicron viruses; nodes represented nucleotide genotypes of viruses and edges between nodes represented pairs of nucleotide genotypes with the highest genetic similarity. We visualized this network using Gephi version 0.9.2 (*20*). We also constructed a phylogenetic tree of those Omicron viruses in NYC using Nextstrain SARS-CoV-2 workflow version 3.0.6 (*21*) and visualized it as timescaled using Auspice version 2.33.0 (https://auspice.us), which is part of the Nextstrain workflow. We then identified different clades of Omicron viruses based on the genotype network and the phylogenetic tree.

To investigate the introductions of the Omicron variant in NYC, we downloaded all global Omicron sequences collected before December 11, 2021 and their metadata from GISAID (https://www.gisaid.org) (*22*). We removed sequences with incomplete information such as collection date or location. We performed mutation calling of these contextual sequences using Nextclade version 1.10.1 (*19*). We applied the same quality control standards for our sequenced samples as we did for GISAID sequences. Sequences that were <29,000 nt long or had Nextclade-assessed QC.overallStatus value below good were considered low quality and removed. To identify the genetic relationship between viruses clustered into different clades from NYC and the rest of the world, we constructed a phylogenetic tree using local viruses and global contextual viruses. We defined the viruses in NYC clustered into these clades as local viruses. For each clade, global viruses detected before the time at which we detected the virus within the clade in NYC were selected as contextual viruses. We used Nextstrain SARS-CoV-2 workflow version 3.0.6 (*21*) to construct this phylogenetic tree and Auspice to visualize it as divergence-scaled.

To investigate the genetic relationship between viruses from travelers and locals, we reanalyzed the same phylogenetic tree that was used to investigate the genetic relationship between Omicron viruses in NYC and highlighted travelers. To reveal the detailed transmission pattern of Omicron in NYC, we analyzed the mutational profiles of Omicron viruses in 2 local districts. In the mutational profiles, we presented only the substitutions that were not Omicron-defining substitutions.

### Data and Code Availability

We have provided GISAID accession numbers and metadata of Omicron sequences generated in this study (Appendix Table 1) and the GISAID global Omicron sequences used in this study (Appendix Table 2). The source code used to generate the figures has been released at GitHub (https://github.com/wuaipinglab/sarscov2-omicron-nyc).

## Results

### Spatiotemporal Distribution of Omicron Variant in NYC

Since the COVID-19 pandemic started in March 2020, we tested 185,870 specimens for SARS-CoV-2 RNA by diagnostic multiplex RT-PCR. A total of 17,058 (9.18%) specimens were positive. These specimens were collected from 87,616 unique persons who were tested once or multiple times. Of those persons, 12,858 had SARS-CoV-2 infection; average incidence rate was 14.68%.

**Figure 1:**
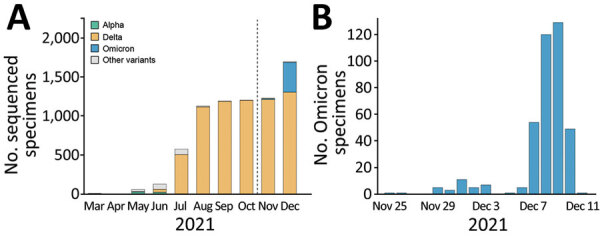
Distribution of SARS-CoV-2 viruses in New York, New York, USA. A) Temporal distribution of 7,237 sequenced viruses, March–December 2021. B) Temporal distribution of 392 Omicron viruses, November 25–December 11, 2021.

A total of 9,516 specimens were run through NGS; 7,237 specimens passed our quality control, of which 392 specimens were identified as Omicron (Figure 1, panel A). Those Omicron viruses were collected during November 25–December 11, 2021 (Figure 1, panel B); they were widespread throughout NYC, and densities were higher in the boroughs of Manhattan and Brooklyn.

### Epidemiologic and Genomic Evidence for Multiple Introductions of Omicron Subclades

**Figure 2:**
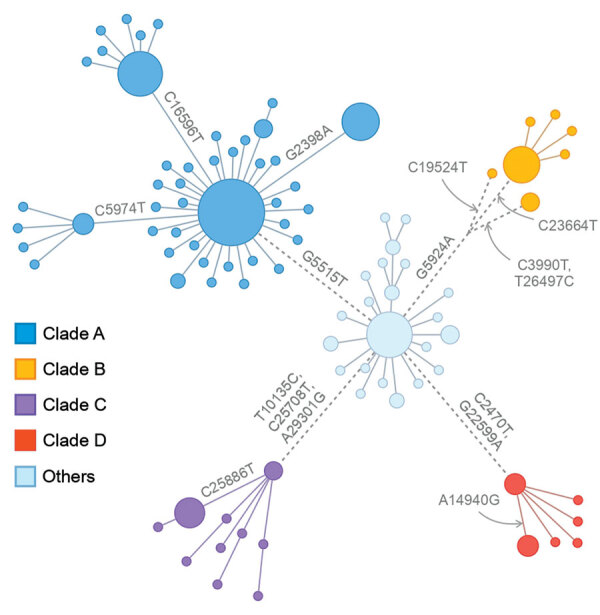
Genotype network of 392 Omicron viruses from New York, New York, USA, November 25–December 11, 2021. In this network, nodes represent nucleotide genotypes by clade, and lines between nodes represent pairs of nucleotide genotypes with the highest genetic similarity. Node size is scaled to log_2_ of the number of viruses with the corresponding genotype. Dashed lines indicate pairs of similar genotypes of different clades.

**Figure 3:**
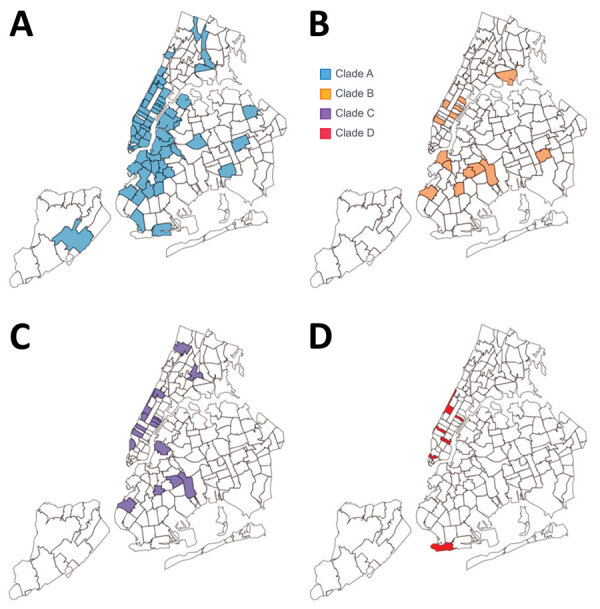
Geographic distributions of 4 main clades of SARS-CoV-2 Omicron variant virus from New York, New York, USA, November 25–December 11, 2021. A) Clade A. B) Clade B. C) Clade C. D) Clade D. Map source: New York City Department of Health and Mental Hygiene (https://github.com/nychealth/coronavirus-data/blob/master/Geography-resources/MODZCTA_2010_WGS1984.geo.json).

**Figure 4:**
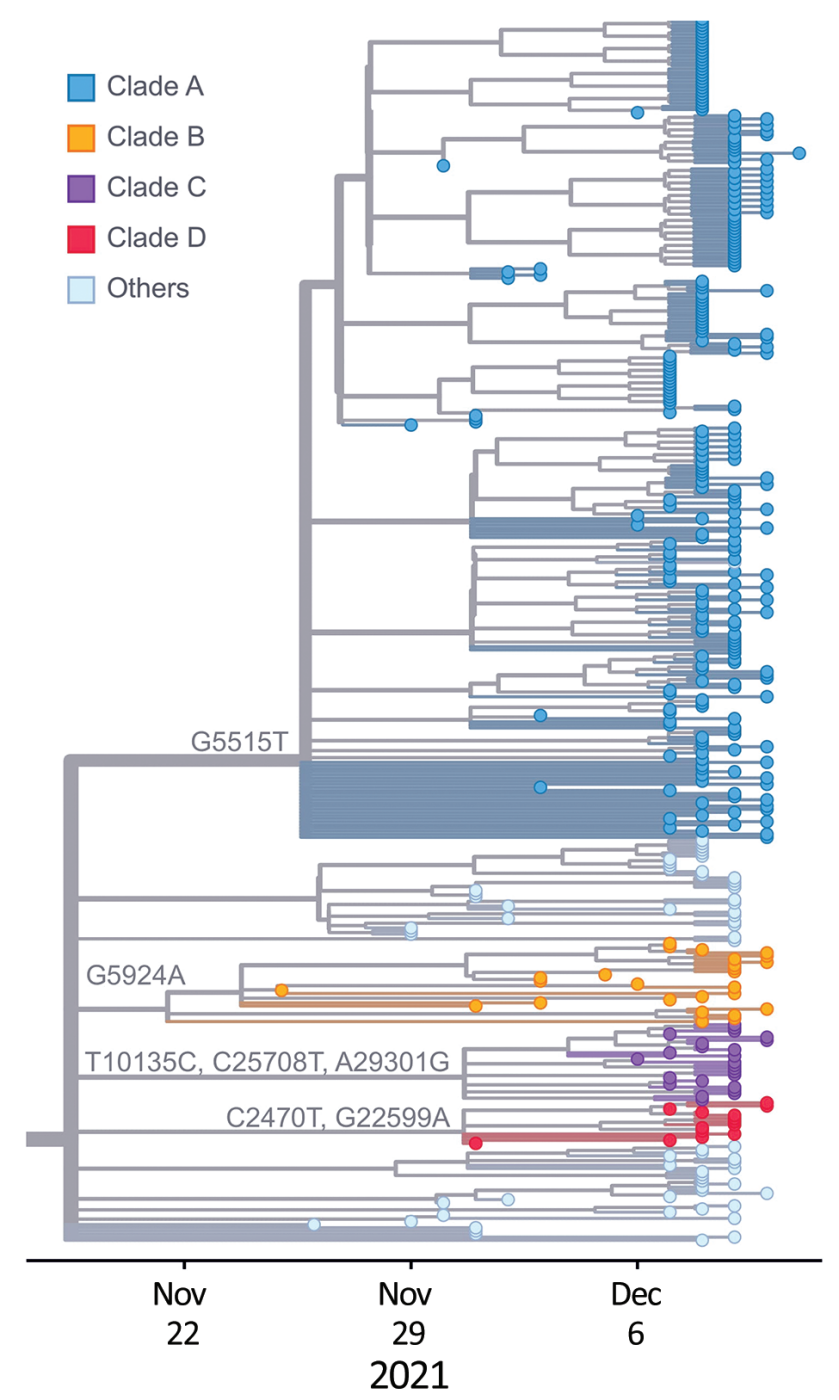
Phylogeny of 392 SARS-CoV-2 Omicron variant virus isolates from New York, New York, USA, November 25–December 11, 2021. Colored dots represent isolates from this study by clade. Substitution locations are indicated.

We performed a phylogenetic analysis of these 392 Omicron isolates. We found that these isolates could be divided into 4 main clades based on the genotype network, phylogenetic tree, and nucleotide substitutions (Figures 2, 3, 4). Most (n = 262; 67%) of these isolates clustered into clade A; those isolates shared a G5515T nucleotide substitution. A total of 65 isolates clustered into 3 smaller clades: clade B (n = 26; 7%), with clade-defining substitution G5924A; clade C (n = 25; 6%), with 3 clade-defining substitutions T10135C, C25708T, and A29301G; and clade D (n = 14; 4%), with 2 clade-defining substitutions C2470T and G22599A. Of note, clade D was consistent with the Omicron subclade BA.1.1 because of its G22599A nucleotide substitution (spike, R346K).

**Figure 5:**
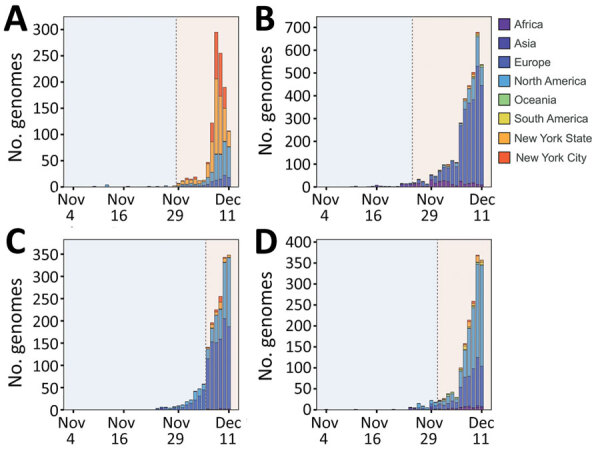
Distribution of SARS-CoV-2 Omicron variant virus isolates clustered into 4 main clades, including viruses identified in this study from New York, New York, USA, November 25–December 11, 2021, and viruses from various regions as obtained from GISAID (https://www.gisaid.org). A) Clade A. B) Clade B. C) Clade C. D) Clade D. For viruses from GISAID, regions were divided into Africa, Asia, Europe, North America (excluding New York state), Oceania, South America, and New York State. Vertical gray dashed lines are to the left of the time at which viruses within the indicated clade were detected in the city of New York during this study. Light blue shading represents the time before our detection of viruses within the indicated clade; light red shading represents the time after we detected the viruses.

We also noted that some Omicron viruses in regions other than NYC had the same substitutions as those 4 clades (Figure 5). To investigate the origins of these 4 clades, we clustered 14,817 global Omicron viruses downloaded from GISAID (*22*) into the 4 clades based on their clade-defining substitutions. Among the global Omicron viruses, 861 (6%) clustered into clade A, 3,563 (24%) clustered into clade B, 1,716 (12%) clustered into clade C, and 1,686 (11%) clustered into clade D.

**Figure 6:**
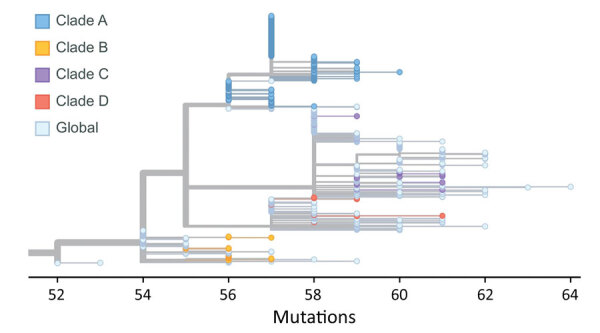
Phylogeny of viruses clustered into 4 main clades, including viruses identified in this study from New York, New York, USA, November 25–December 11, 2021, and contextual viruses in various regions from GISAID (https://www.gisaid.org). For each clade, we designated global viruses detected before the time at which we detected the virus within the clade in New York as contextual viruses for phylogeny construction. Colored dots represent viruses from New York by clade; light blue dots represent global contextual viruses.

We subsequently investigated the spatiotemporal distribution and phylogenetic relationship of global and NYC viruses within these 4 clades (Figures 5, 6). Clade A and its corresponding substitutions were initially detected in NYC on November 29, 2021; clade B on November 25, clade C on December 6, and clade D on December 1. Of note, we found viruses from other regions clustered into these 4 clades that had been collected earlier than our sequenced viruses in NYC (Figure 5): a total of 12 clade A viruses, 71 clade B viruses, 233 clade C viruses, and 83 clade D viruses. The earlier detection of these viruses in other regions suggests independent introductions into NYC.

To further identify the potential introductions, we constructed a phylogenetic tree using viruses clustered into clades A, B, C, and D, including 327 viruses from NYC and 399 viruses from around the world (Figure 6). We found that the early viruses clustered into clade A were detected in North America, Africa, and Europe. Of note, 3 of them (GISAID accession nos. EPI_ISL_7129868, EPI_ISL_7782594, and EPI_ISL_7908023) were detected in other laboratories in New York state. In the phylogenetic tree, early global clade A viruses were located near the base of clade A, and some viruses in NYC were distributed along main branches. Combining the phylogenetic and spatiotemporal distribution of all clade A viruses, we inferred that this clade was introduced into NYC and then spread through local transmission.

Most clade B early viruses were detected mainly in Africa, with sporadic detection in Europe, North America, and Asia. The viruses in NYC clustered closely with those viruses in the early stage of the wave. Clade B viruses were detected only in Africa before November 21, 2021, suggesting that clade B had spread outside of Africa after early local transmission. Thus, we believe that clade B viruses in NYC were the result of another independent introduction. Similarly, clade C and clade D were distributed in regions including Europe, North America, and Africa before our detections in NYC. The close genetic relationship within these 2 clades suggests 2 additional independent introduction events.

### Potential Importation Risk for Omicron Variant from Travelers

**Figure 7:**
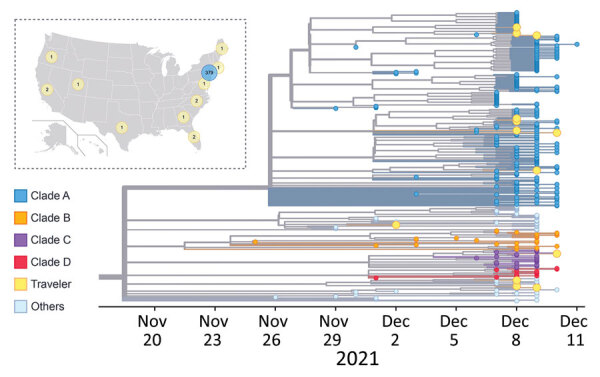
Phylogeny of SARS-CoV-2 Omicron viruses identified from travelers and locals in this study, New York, New York, USA, November 25–December 11, 2021. In the phylogenetic tree, colored dots represent viruses from New York residents by clade. Yellow dots represent viruses from travelers. The inset map shows the states and number of patients with isolated viruses. Yellow circles represent travelers’ home states; blue circle represents local New York residents. Map source: Wikimedia Commons (https://commons.wikimedia.org/wiki/File:Blank_US_Map_(states_only).svg).

Our phylogenetic analysis shows that the Omicron variant outbreak in NYC likely resulted from multiple independent introductions. We found that, among the 392 sequenced Omicron viruses in NYC, 13 of them were obtained from domestic travelers from the following states: California (2), Florida (2), Georgia (1), Maryland (1), Maine (1), North Carolina (2), Oregon (1), Rhode Island (1), Texas (1), and Utah (1) (Figure 7). To investigate the genetic relationship between viruses from travelers and locals, we analyzed the 392 Omicron viruses in a timescaled phylogenetic tree, which we constructed using Nextstrain workflow and visualized as timescaled using Auspice (*21*) (Figure 7). The viruses from travelers were distributed across the phylogeny: 8 of them fell into clade A and 1 into clade C; the remaining 4 did not fall into any of the 4 main clades being discussed. Considering the collection time and genetic similarity, we did not find irrefutable evidence that the sequenced viruses from domestic travelers were the origin of the Omicron variant in NYC.

### Community Transmission and Co-circulation of Omicron Subclades in NYC

The Omicron variants were distributed in various areas in NYC (Figure 3). To investigate if introductions from outside NYC led to local transmission, we constructed the genotype network and timescaled phylogenetic tree of these 392 viruses (Figures 2, 4). We constructed this phylogenetic tree using Nextstrain workflow and visualized it as timescaled using Auspice (*21*). In addition to clade-defining substitutions, we found many novel heritable substitutions added to the subsequent progenies. The close genetic relationship within different clades showed that multiple clades spread in NYC with a co-circulation pattern after introductions.

**Figure 8:**
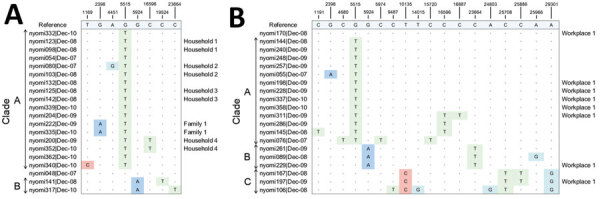
Mutational profiles of SARS-CoV-2 Omicron variant viruses in 2 local districts, New York, New York, USA, November 25–December 11, 2021. A) District of postal (ZIP) code 11201. Viruses isolated from patients within the same living address or the same family are labeled on the right. B) District of ZIP code 11220. Viruses isolated from patients working at the same building are labeled on the right. Column labels at top indicate substitution locations; shading indicates substitution from any nucleotide to a nucleic acid: dark blue indicates substitution to adenine, red, substitution to cytosine; green, substitution to thymine; and light blue, substitution to guanine.

**Figure 9:**
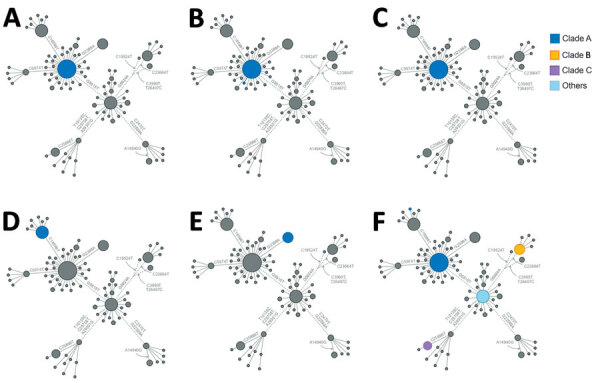
Genotype network mapping of transmission events of SARS-CoV-2 Omicron variant in specific groups, New York, New York, USA, November 25–December 11, 2021. Colored dots indicate genotypes of viruses within each transmission event by clade, if known. A) Household 1. B) Household 2. C) Household 3. D) Household 4. E) Family 1. F) Workplace 1.

We also discovered several instances of Omicron community transmission in NYC. We noted the viral distribution in 2 postal (ZIP) codes that had the highest detected number of viruses (Figure 8). In the 11201 ZIP code, we found the 2 viruses (nyomi222 and nyomi335) shared the G2398A substitution that belonged to 2 patients from the same family (family 1). Two viruses (nyomi200 and nyomi352) from patients living in the same household shared the C16596T substitution (household 4). These viruses with shared genome sequences from patients within the same family or the same household address suggest local household transmission (Figure 8, panel A; Figure 9). In the 11220 ZIP code, 8 viruses were from patients working in the same building (workplace 1), and 4 of them (nyomi198, nyomi228, nyomi337 and nyomi358) shared identical genetic substitutions. This epidemiologic evidence suggests community transmission in the workplace (Figure 8, panel B; Figure 9). We also found that even viruses isolated from patients at the same working address might fall into different clades, implying complex and possibly cryptic transmission.

## Discussion

Since the first detections in November 2021, the Omicron variant has gained global attention for its increased transmissibility and immune escape (*23*). It was identified in 87 countries within 3 weeks (*2*) and established itself as the global dominant variant within a few months (*3*). Although several measures were implemented to reduce the introduction and spread of SARS-CoV-2 into the United States, 22 states reported >1 case of Omicron as of December 8, 2021 (*24*). As a major cosmopolitan city, NYC has been challenged with multiple introductions of different variants of SARS-CoV-2 during the pandemic (*13*–*15*). The high contact rates observed in densely populated cities may promote community transmissions of the virus (*25*).

Our analyses suggest that there have been 4 main independent introductions of different Omicron subclades into NYC from regions including Africa, Europe, and North America during the early outbreak of Omicron. Those introductions were followed by subsequent community transmission across NYC. Similarly, the rapid local spread after early Omicron introductions was observed in Finland (*10*), Denmark (*11*), and Mexico City (*12*). In addition, through the combination of genome sequencing analysis and epidemiologic studies of SARS-CoV-2 patients in individual districts of NYC, we discovered evidence of both household and workplace transmission patterns. Our observation of multiple Omicron introductions followed by onward transmission during a 17-day period highlights the potential for introductions of emerging variants to spread locally. Therefore, after emerging variant outbreaks, timely enhanced tracking and monitoring of travelers and subsequent transmission reduction interventions are urgently needed to ensure that those introductions do not result in widespread community transmission.

Given the limited sequencing coverage and surveillance, we were unable to determine the first case of the introduction for each of 4 clades in our study and its subsequent transmission chain. Differences in sampling and sequencing may bias results and make accurate estimation of introduction times difficult. In addition, the possible convergent evolution of viruses within immunocompromised or immunocompetent hosts in NYC could complicate interpretation. As more genomes of Omicron viruses are being sequenced, we expect to be able to further elucidate the origins of Omicron introductions and chain of the community transmission.

Our analysis also highlights the importance of timely genomic surveillance, which can reduce the effects of emerging variants (*26*). Through genomic surveillance, we can make an initial assessment of the risk for the emerging variant by its mutation profile and growth advantage (*2*). Analysis of sequenced data can augment testing strategies to monitor the variant in real-time without whole-genome sequencing. For example, SGTF can be used as a proxy for Omicron during the early days of an outbreak (*8*–*10*). In addition, the introduction and transmission pattern of the emerging variants can be elucidated using viral sequences. Early identification of the emerging variant outbreak provided by genomic surveillance could aid us in making timely and appropriate policy responses, including enhanced tracking and monitoring of travelers and social control measures. Therefore, combining real-time genomic and epidemiologic surveillance is critical for effective responses for tracking, understanding, and controlling infectious disease outbreaks.

Appendix Additional information about early transmission of Omicron variant of SARS-CoV-2 virus, New York, New York, USA.
